# A crowding free digital interface to help French-speaking children learn to read

**DOI:** 10.1371/journal.pone.0323623

**Published:** 2025-06-25

**Authors:** Viet Chau Linh Nguyen, Guillaume Lio, Thomas Perret, Alice Gomez, Angela Sirigu

**Affiliations:** 1 Institute of Cognitive Science Marc Jeannerod, Centre National de la Recherche Scientifique, Bron, France; 2 Le Vinatier Hospital, Bron, France; Federal University of Paraiba, BRAZIL

## Abstract

Learning to read is a challenging task for first-graders. Letter crowding in the peripheral visual field has been identified as a key interference process during reading acquisition. To reduce crowding and enhance selective attention, we designed a new way to read (Digit-tracking) in which words and sentences appear blurred. By sliding the index finger along the blurred text, the letters just above the finger position appear unblurred and are seen in foveal vision. We hypothesized that this approach might facilitate orthographic decoding and promote reading skills. Using a tablet device, two groups of first-grade children (N = 54) were trained on digit-tracking exercises and paper exercises using a crossover design. Results showed that performance in letter, syllable and meaningless text-reading was significantly higher after digit-tracking training compared to paper-based training. Using the recorded finger trajectories as a proxy for eye movements, we found that text scanning patterns (saccade length, landing position, regressive saccades) predicted children’s decoding and fluency. We conclude that training with the digit-tracking procedure accelerates decoding and reading fluency in school beginners and may provide a sensitive metric of reading competence.

## Introduction

Learning to read is difficult for beginning readers, and poor readers usually experienced reading difficulties in their childhood [1–3]. Attention and executive control are critical functions for early reading acquisition [4], as children struggle to inhibit exogenous and endogenous distracting stimuli [5]. As attention and inhibitory control are still immature in beginning readers [6], teachers need to foster these skills on a daily basis or offer opportunities to circumvent the immaturity of these processes. For example, the simple gesture of pointing to words by an adult while reading a book increases attention in 3- to 5-year-olds, and this effect is reinforced at age 5 [7]. Other findings also show that the teacher’s pointing gesture during reading increases the likelihood that children will imitate this behavior [8], providing an opportunity to spontaneously direct attention [9]. In addition, it has been shown that finger pointing to a previously encoded text (e.g., a text read several times by parents), seems to improve children’s understanding of how to decode the text units [10]. Finally, several studies have demonstrated that haptic exploration itself involves a multisensory integration process between visual and auditory stimuli and could help in learning the letter-sound association [11–13]. In summary, pointing to the printed text by the beginning reader could be a relevant strategy to signal the need for attentional control and to promote learning of letter/sound correspondence through an embodied process.

Another challenge that children face while learning to read is the reading material *per se*. Reading strings of letters, syllables and words is difficult because of the crowding effect, a process in which nearby visual elements interfere with central vision and result in a decreased ability to identify the target [14]. Visual crowding has a major impact on reading quality and is linked to slow reading and dyslexia [15–18]. Crowding intensity is modulated by eccentricity: the farther a stimulus is from the fixation center, the more difficult it is to identify [19]. Visual processing in extrafoveal -which includes both parafoveal (2° to 5° from the fixation center) and peripheral (>5°)- vision is therefore particularly vulnerable to the crowding phenomenon [20,21]. Sensitivity to crowding in pre-readers predicts future reading difficulties [18] which can be alleviated by letter spacing [22].

In most reading contexts, skilled readers extract useful information in the parafovea [23,24], and this benefit outweighs the cost of preview processing generated by extrafoveal crowding [25,26]. However, because of the crowding effect, parafoveal information does not benefit novice readers [27]. This leads to the hypothesis that in readers with poor decoding skills, learning to read can be facilitated by suppressing parafoveal information in order to ease the decoding process.

Previous studies support the facilitatory effect of reducing crowding during reading in vulnerable populations using a variety of methods (inter-letter spacing, word by word presentation, etc.) [28–34]. The cost of crowding can also be reduced by a pointing gesture on the text to enhance attention on relevant items [35], an approach similar to cueing in crowding contexts [34]. Although relevant, these observations have not yet led to the development of reading devices for young beginning readers.

One way of stimulating attention to the foveal field and reducing crowding is to design tools that facilitate visual tracking by masking parafoveal information. Here, we use a digital tool based on a method called digit-tracking previously employed to study visual attention and images exploration in neurotypical subjects and in autism spectrum disorders [36]. The procedure involves presenting blurred images on a tablet, processed with a Gaussian filter to mimic the low spatial resolution of the peripheral retina. By sliding the index finger on the screen, a portion of the image of the size of the foveal area appears in full resolution. The attention maps computed with this method are highly correlated with those derived from classical eye-tracking techniques, showing that digit-tracking procedure can serve as an indirect measure of eye movements. Applied to reading, this method improves decoding and accelerates reading acquisition by reducing the crowding effect and reinforcing children’s attention on target letters.

The aim of the present study was therefore to determine if reduction of crowding and focused orthographic attention with digit-tracking facilitates the acquisition of decoding and reading fluency of first-grade French school-children.

Another interesting feature of this method is the recording of finger movement trajectories which provides an indirect but reliable measure of eye movements [36–38]. Studies investigating eye movements trajectories during reading have shown that proficient readers make fewer, shorter fixations, longer forward saccades, and fewer backward saccades compared to beginning readers [23,24,39–42]. They have also shown that the first fixation falls in a spatial position that maximizes the visibility of all letters in the word [43,44]. By the end of first grade, children fixate at a position near to that of adults [45,46] but closer to the beginning of the word [47] in addition to produce more re-fixations on long words [47,48]. Thus, eye movements can provide valuable information for assessing the evolution of reading skills during the first school year, although the technical constraints associated with eye-tracking devices have made their use in the classroom difficult. Using the digit-tracking method, we intend to overcome this difficulty and provide a new tool for examining children’s reading performance in a minimally intrusive manner. Hence, just as revealed by eye movements recording, we expect to reproduce a blueprint of children’ reading abilities with the analysis of digital movements (shorter digital fixations, longer forward digital “saccades” and fewer backward digital “saccades” should differentiate good from poor readers).

## Materials & methods

### 1. Participants

A power analysis was conducted using G*Power [49] for a within- and between-subjects design. Since no previous study has employed the specific procedure used in our experiment, we could only estimate the likely effect size. To ensure the most conservative approach, we opted for the smallest possible effect size (f = 0.10) with an alpha level of 0.05. The analysis indicated that a total sample size of 44 participants would be required to achieve a power of 0.80. In order to guarantee a larger sample size due to potential drop-out and because parental consent forms from each class were collected simultaneously shortly before the study started, we included a larger number of participants, resulting in a total of 58 participants.

Fifty-eight first-graders (30 girls) from a French public primary school in Lyon were included in our study. Children’s mean age at the time of the first evaluation in November 2018 was 6.3 (SD ± 0.3, range 5.9–6,8). Among the initial 58 children, four were excluded: three in the first training phase, because they did not comply with the task instructions due to lack of motivation or understanding, and one at the end of the first training phasedue to school withdrawal. Hence, data of 54 children were kept for analysis. All children had normal or corrected-to-normal vision and none had hearing loss. The study was conducted in accordance with the Declaration of Helsinki. Parents were informed of the purpose of the study and gave their written informed consent for the child to participate in the investigation. The experimental procedures were approved by the French National Ethic committee on February 2018 (CPP n° 3574, 2017-A03065-48). Prior inclusion each parent signed an informed written consent where they agreed for their child participation in the study. The inclusion started on November 12, 2018 and ended on February 2019. All families received information from the French Ministry of Education regarding the national school evaluation procedure and agreed to the European regulation procedures on data protection.

### 2. Procedure

**Fig 1:**
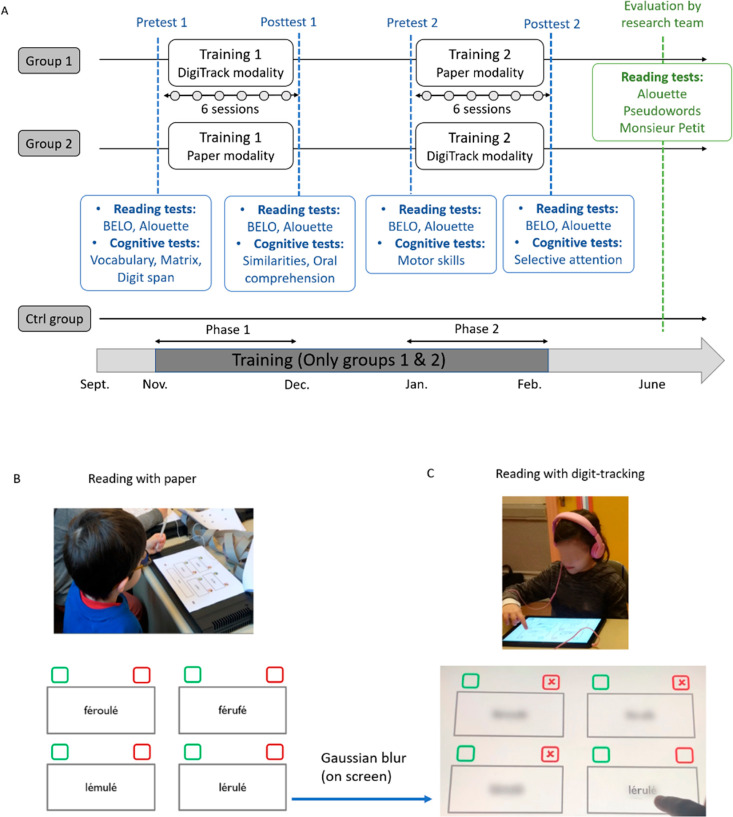
Study Design. (A) First-grade children learned to read in experimental training sessions with digit-tracking or paper-and-pencil reading exercises (N = 54). During the first training phase Group 1 started the exercises on the digit-tracking modality and Group 2 on the paper modality. Then, each group switched to the other modality during the second phase. Training material in both modalities involved the same exercises. Each training phase comprised 6 training sessions of 35 minutes each. The experimental groups were assessed before and after each training phase on *decoding and reading fluency* (Letter and syllables reading assessed by BELO test, and meaningless text reading assessed by Alouette test). General cognitive abilities were assessed once throughout the training period. At the end of the school year, reading scores of children trained during the previous phases along with 19 other children who did not participate in training (Control group) were assessed using three tests: pseudowords reading, Alouette test (for meaningless text reading) and Monsieur Petit (for meaningful text reading). (B) An example of a child participating in a reading exercise with the paper modality and (**C)** with digit-tracking modality. Photos were taken by Alice Gomez.

The study consisted of a pre-post training evaluation using an experimental cross-over design for modality effects: Digit-tracking versus Paper. Two experimenters trained the children who were randomly assigned to one of the two experimental groups: Group 1 began with a training phase in the digit-tracking modality followed by a training phase in the paper modality; Group 2 was trained first in the paper modality and then in the digit-tracking modality (see Fig 1). The use of cross-over design was motivated by the fact that it reduces the impact of inter-individual differences, evaluates the efficacy of introducing the digit-tracking training at different points in the learning process and allows each child to receive training with the digit-tracking modality. We assessed children’s decoding and reading fluency at the start and end of the experimental training phases (Digit-tracking/Paper) (see Fig 1), as well as their general cognitive abilities.

The whole experiment took place on the school’s site which provided a dedicated space to conduct the experiment. A single room was devoted to individual testing while two other rooms were set up for group experimental training sessions. Five experimenters collected the data. For each child, the same experimenter collected the data during the entire protocol with fixed task order. For computerized tasks, children were seated in front of a Dell latitude tablet (1920 x 1280 pixels) laid on a table at a viewing distance of 30 cm. The sampling rate was 60 Hz. The tablet’s monitor was calibrated using the default gamma correction value (2.2), where the signal is adjusted to ensure the image appears natural. For the paper material, we used a printer with standard gamma correction, ensuring consistency between the materials displayed on the tablet and on paper. We used standard plain, non-glossy paper to limit light reflection. A visual comparison under daylight revealed no differences between the two materials in terms of color brightness, contrast, or detail accuracy.

All children received a book as a reward for their participation.

#### 2.1. Neuropsychological and reading evaluation.

2.1.1. ***General cognitive abilities: group 1 vs group2***. To ensure the homogeneity of the experimental groups (group 1 and group 2), we controlled for age, gender, intelligence level (matrix subtests of WPPSI battery, [50], selective attention (flanker test, [51], motor (NEPSY II, [52], reading (Vocabulary, from the WPPSI, [50] and oral comprehension, ELO, [53] skills. We found no significant differences between group 1 and 2 in general cognitive abilities (all *p*s > 0.09, see Table 1) although the difference on matrix scores was marginally significant (p = 0.09) where group 2 showed better fluid reasoning abilities than group 1.

**Table 1:** Mean and standard error (SE) of cognitive abilities in group 1 (N = 27) and group 2 (N = 27), on the subtests from the WPPSI, (vocabulary, similarities, matrix, cancellation, [50]), ELO, (Oral comprehension, [53]), WISC-IV (Digit span, [54]), Flanker test [51], NEPSY-II (motor skills, [52]). WPPSI and NEPSY-II scores are standardized (mean of the population for this age range is 10, SD = 3). For the ELO comprehension test, the mean of the first-grade population is 19.2, SD = 1.7). For the Flanker test, we report the time difference in milliseconds between congruent trials and incongruent trials. No significant difference was observed across groups. P-values from 2-samples T-tests for normal distributions or Wilcoxon if one of the two distributions are not normal (#) – normality being assessed using the Shapiro test.

General cognitive abilities test	Group 1	Group 2	*p*-values 2 samples t-test or Wilcoxon test (#)
	Mean *(SE)*	Mean *(SE)*	
Vocabulary	12.41 *(0.73)*	13.65 *(0.63)*	0.21 #
Similarities	10.97 *(0.76)*	10.96 *(0.55)*	0.99 +
Oral comprehension – ELO battery	17.690 *(0.46)*	18.08 *(0.34)*	0.61 #
Matrix	9.76 *(0.60)*	11.19 *(0.60)*	0.09 +
Digit span (forward)	12.69 *(0.61)*	12.39 *(0.54)*	0.71 +
Digit span (backward)	10.96 *(0.54)*	9.73 *(0.41)*	0.11 +
Selective attention (ms)	93.833 *(29.70)*	151.55 *(25.67)*	0.13 #
Cancellation	9.66 *(0.46)*	10.27 *(0.54)*	0.42 #
Motor skills	12.41 *(0.52)*	11.92 *(0.68)*	0.56 +

2.1.2. ***Reading assessment***. Reading abilities were also assessed through letter and syllable naming and meaningless text reading before and after each training phase in both experimental groups.

***Letter and syllable naming.*** We used a subtest of the BELO (*Batterie d’évaluation de l’écriture et de l’orthographe)* test [55], in which children were presented with a set of orthographic elements (a, u an, on…) t instructed to read aloud. After five consecutive incorrect responses, the task was discontinued. The total score is computed as the total number of elements correctly read, with a maximum score of 85 (see S1.Text in S1 File).

***Meaningless text reading***. Children were tested with the French Alouette test [56], classically used to assess children’s lexical abilities and to detect dyslexia. A paper sheet text containing 265-meaningless, words was presented and children were asked to read aloud as accurately and quickly as possible during three minutes (See S1.Text in S1 File).

We found no significant group differences between the baseline scores (at Pretest 1) for letter and syllable reading (Group 1: mean = 47.14, *SE = *3.30; Group 2: mean = 47.15, *SE = *3.48; F(1,104) = 0.14, *p *= 0.71), meaningless text reading fluency (Group 1: mean = 9.46 words/min, *SE = *1.70; Group 2: mean = 11.70 words/min, *SE = *3.02; F(1.105) = 0.52; *p *= 0.47) and meaningless text reading accuracy (Group 1: mean = 65.23%, *SE = *3.35; Group 2: mean = 64.10%, *SE = *3.46; F(1, 104) = 0.01, *p *= 0.94).

2.1.3. ***Final evaluation in June***. Reading abilities were also assessed four months after the end of the intervention using classical reading task of pseudowords and of meaningful and meaningless texts. Pseudowords reading: Children were presented with a sheet with the pseudo word in column printed in Times New Roman (size 16) and spaced. They were asked to read the word aloud as quickly and accurately as possible. Forty-five pseudowords were selected from the pseudowords sample used by Bosse and colleagues [57]. Meaningful text: Using the Monsieur Petit French standardized reading fluency test [58] the children were shown a sheet paper with the printed text, and asked to read it as correctly and quickly as possible during 1 minute (see S1.Text in S1 File). Meaningless text reading: Using the French standardized reading test “Alouette R” version [56], children were asked to read a printed meaningless text as quickly and accurately as possible, within 3 minutes time-limit (see S1.Text in S1 File).

#### 2.2. Experimental training through reading exercises.

The two experimental groups (group 1 and 2) underwent two training phases each lasting 4 weeks and comprising 6 sessions. Each training session led by an experimenter involved a group of up to 5 children and lasted 35 minutes. Within this small group each child completed the exercises alone. At the beginning of each new exercise, the experimenter explained the procedure. When individual questions were asked about the exercise, the experimenter gave collectively the correct answer. The children were then invited to move on to the next exercise by touching the arrow at the top of the tablet screen (group 1) or by turning the page of the paper booklet (group 2).

To make the training pleasant and playful children performed four types of reading exercises: 1) a pseudo-words visual matching 2) syllables visual-auditory matching 3) pseudo-words auditory-visual matching 4) sentences reading comprehension. In the pseudo-words visual matching exercise, children saw a written target pseudo-word (e.g., “rufolé”) at the bottom of the screen and had to select among the 4 written pseudo-words (“RUFOFÉ, RUFOLÉ, RUFALÉ, MUFOLÉ”) at the top those that matched the target (the target was in lowercase whereas the stimuli to be selected were in uppercase to avoid matching based only on features similarities and ensure that the children made good choices based on letter knowledge). In the syllables visual-auditory matching task, children read a written target syllable (e.g., “MI”) at the bottom of the screen and had to select among the 4 drawings (e.g., a drawing of an ant, in French: “FOUR**MI**”, a stamp, a motorbike, a strawberry) from the top, which one contained the target sound. In the pseudo-words auditory-visual matching, children heard a target pseudo-word (e.g., “falaflu”) and had to select among the 4 written pseudo-words (“falaflu, lafafu, feleflu, folaflu”) at the top, those that matched the target sound. In the sentence comprehension exercise, children had to read a complete written sentence (e.g., “LE CHAT EST SUR LA TABLE”, *the cat is on the table*) and then had to choose which among the 4 drawings, at the bottom, best matched the sentence (See Fig 1B for more details on these exercises).

Across the two training phases, stimuli were paired in a way that the syllabic structures of pseudo-words were similar between the first and the second phase (See S1.Table in S1 File). The phonemes and syllables chosen to construct pseudo-words were those that children were already taught in class until the week preceding the training session. Hence, as children went to more advanced training session, we introduce progressively new phonemes and syllables that they have been taught recently by their teachers. For trials involving sentences, we built sentences that had a percentage of phonemes decodable higher than 60% at the moment of training using the Anagraph platform [59]. In the first three sessions, children completed 10 written pseudowords (WPW) trials, 10 syllables (Sy) trials and 10 oral pseudowords (OPW) trials. For the last three sessions, they performed 10 WPW trials, 5 Sy trials, 5 *SE* trials and 10 OPW trials.

2.2.1. ***Paper modality***. Both groups performed half of their training under the paper modality. In this modality, the exercises were color-printed on an A4 booklet provided to each child (one booklet per session per child) with an exercise per page. Booklet were laid on a table at a viewing distance of 30 cm.

2.2.2. ***Digit-tracking modality***. Both groups completed half of their training in the digit-tracking modality. They received the exercise on a tablet with the digit-tracking interface that blurred the written texts and allowed children to unblur the text by pointing to the relevant area with their finger.

An algorithm constructed using the Psychtoolbox from the Matlab Software (version 8.1) allowed to simultaneously present stimuli and collect finger touch exploration data (time and coordinates). Stimuli were presented on a Dell latitude tablet (1920 x 1280 pixels) laid on a table at a viewing distance of 30 cm. The sampling rate was 60 Hz. Written elements (syllables, words, pseudo-words, sentences) were blurred using a Gaussian blur filter with a standard deviation of 20 pixels. After the blurred stimulus appeared, by touching the screen with the finger, a window appeared where the original (unblurred) text was displayed is in clear.

To rule out the possibility of a motivational effect driven by the use of the digital device, a comparison between training with paper modality and training with a tablet modality without digit-tracking was carried out in a preliminary phase in another group of children (mean age = 5.68, min = 5.22 max = 6.70 – See S2.Text & S2.Table in S1 File). We observed no difference between these two modalities on changes in different reading-related abilities before and after the training (letter recognition: *p *= 0.90; *p*honemes recognition: *p *= 0.57; syllables recognition: *p *= 0.959, see S3.Fig in S1 File; sound recognition: *p *= 0.57; word recognition: *p *= 0.22).

#### 2.3. Digit movement data.

2.3.1. ***Pre-processing.*** Data were pre-processed and analyzed using homemade Python 3.7 scripts [60]. When children were trained with digit-tracking (training 1 of group 1 and training 2 of group 2), we collected all finger movement coordinates every 16.7 ms (sampling rate: 60 Hz). Coordinates collected as percentage of screen (width for X-coordinates and height for Y-coordinates) converted into coordinates pixels. For each child and session, the median movement speed was computed (after excluding all null values) from all collected data points and defined as the individual threshold. The median speed threshold was then used as a cut-off to define *digital fixations* (i.e., when the finger moves slower than the median speed of the subject) and *digital saccades* (i.e., when the finger moves faster than the median speed of the subject). We chose the median speed as threshold value empirically: across a large range of speed thresholds, this value detected the highest number of fixations. The *digital fixation duration* is computed as the total duration in milliseconds before the next digital saccades appears; the *position of fixation* (X, Y) is computed as the barycenter of all the position belonging to this digital fixation (that is with a maximum distance in X lower than 5.8 pixels and in Y lower than 6.4 pixels); the *number of digital fixations* is computed by counting the number of streak of contiguous points the finger moves slower than the median speed during the trial or over the stimulus in the region of interest. The *digital saccade length* is the distance between two consecutive fixations, computed as the differences between coordinates (X,Y) of the fixation at the end and at the beginning of the saccade (in millimeters). The *digital saccade speed* is computed as the average speed in each point belonging to the saccade and expressed in millimeters per second (with the points belonging to the saccade defined according to the median speed threshold). A saccade was defined as progressive if the X-coordinate of the last point is greater than the X coordinate of the first point and regressive otherwise. The *proportion of digital regressive saccades* was computed as the ratio of the number of regressive saccades per total number of saccades for each trial of each subject.

2.3.2. ***Density maps construction***. For sentences, we plotted density maps of first fixations on words using ggplot2 package [61] of R software (version 3.6.2, R Development Core Team, 2018). For each word, the X-coordinate of the first fixation for each individual was computed, then, X-coordinates from each individual were pooled according to the group (good or bad decoders) and smoothed using 0.4 time the default bandwidth provided by the package, available at https://www.rdocumentation.org/packages/stats/versions/3.6.2/topics/bandwidth. Finally, for better visibility, values are multiplied by the number of subjects in the group.

#### 2.4. Statistical analysis.

All statistical analyses were performed using R software (version 3.6.2) [62]. Parametric statistical tests were conducted after ensuring that data did not violate the following conditions: normality and homoscedasticity of residuals. All statistical tests are two-sided.

2.4.1. ***Impact of digit-tracking vs paper modality.*** For reading analysis, we computed the *accuracy,* that is the percentage of items correctly read, the *fluency*, that is the number of items correctly read per minute (not in the syllables and letter test), in each reading test (pseudowords, meaningless text and meaningful text reading) and the *change* in the percentage of letter and syllable reading by calculating the gain between the pre and post-test score as reading dependent variables. All non-reading general cognitive abilities presented in Fig 1A, as well as age, were added a priori as covariates in our LMMs for assessing reading change.

### Model validation: AIC, BIC, and RMSE analysis

To validate our linear mixed models (LMMs) and assess the robustness of the results, we compared the models using three complementary criteria: Akaike Information Criterion (AIC), Bayesian Information Criterion (BIC), and Root Mean Squared Error (RMSE). These metrics provide a thorough evaluation of model performance, helping to ensure an appropriate balance between model complexity and goodness of fit.

Before including general cognitive abilities as covariates, we compared the AIC of the model with the minimal model (without covariates). In every case, the full model, which accounted for modality, phase, and cognitive variables, showed the lowest AIC value (598.55) compared to the minimal model (AIC = 777.26). Based on these results, the following linear mixed model was used:


$$\begin{array}{cc} \text{Reading\textbackslash{} change}\;\text{variable}\; & \sim \;\text{Modality\textbackslash{} *Phase\textbackslash{}, + \textbackslash{}, Age\textbackslash{}, + \textbackslash{}, Similarities\textbackslash{}, + \textbackslash{}, Matrix\textbackslash{}, + \textbackslash{}, Vocabulary}\;\\ & +\;\text{Oral\textbackslash{} comprehension\textbackslash{}, + \textbackslash{}, Memory\textbackslash{} span}\;\\ & \text{+ \textbackslash{}, Cancellation\textbackslash{}, + \textbackslash{}, Selective\textbackslash{} attention }+\;(1|\text{subject})\;+\;\in . \end{array}$$

In addition to AIC, we also computed BIC and RMSE to further validate our models. For letter and syllable reading (BELO test), the full model incorporating general cognitive abilities had a lower BIC (634.638) compared to the minimal model (BIC = 793.24), suggesting a better fit, although it exhibited a slightly higher RMSE (11.47 vs. 10.88 for the minimal model). For meaningless text reading fluency (Alouette test), a similar trend was observed. The full model showed a superior fit with a lower BIC (526.57) than the minimal model (BIC = 619.71), while the RMSE values were nearly identical (6.73 for the full model vs. 6.64 for the minimal model). For meaningless text reading accuracy, the full model again demonstrated better performance based on both BIC (656.15) and RMSE (9.50), compared to the minimal model (BIC = 797.44, RM*SE = *9.65). Since BIC and AIC both penalize models with more parameters, their consistently lower values for the full models indicate that they offer a better balance between complexity and fit, reducing the risk of overfitting. Although RMSE differences between the full and minimal models were minimal, the overall goodness-of-fit statistics support the use of the full models for the training modality effects. These validation checks demonstrate that our models, incorporating cognitive abilities as covariates, provide the best fit to the data. This approach strengthens the robustness of our conclusions regarding the effects of the training modalities.

We used the *lmer* function from the *lme4* package (version 1.1.-10) [63] with modality (digit-tracking vs Paper) and training Phase (1 *vs.* 2) as fixed effect, participant as random effect and other cognitive effects as parametric variables. For digital movement analysis, we analyzed the digital fixation duration, the first digital fixation position, the number of digital fixations, the digital saccade length and speed as finger movement dependent variable.

### Effect sizes calculation

When using linear mixed models (LMM), there is no consensual way to calculate standard effect size similar to Cohen’s *d* for main effects or interactions. We followed Pek & Flora ‘s guidelines [64] by reporting unstandardized effect sizes *b* in our models. This value represents the difference of interest in the original unit of measure, *e.g.,* change in BELO score, Alouette fluency, or finger kinematics measures, which, according to Pek & Flora, is more meaningful than a unitless standardized effect size.

However, we also reported values of R², which is the equivalent of η² for mixed models. This value accounts for the proportion of variance explained by the model (referred to as R²_model_) or by a given fixed effect (referred to as R²_X_, with X = the variable of interest). This value was obtained using the function *r2beta* from the *r2glmm* R package [65], and using the proportion of variance explained by the fixed predictors. This method was introduced by Nakagawa and Schielzeth and later modified by Johnson [66,67].

2.4.2. ***Final evaluation in June.*** Since the LMM is only used in repeated or clustered observations [68], these data with only one value per subject was analyzed using ANOVA.

2.4.3. ***Finger movement data***. Finger movements during reading were analyzed using LMM for robustness and statistical power optimization with unbalanced and incomplete data. LMM is a more suitable tool than repeated measures ANOVA in some instances, such as missing data [69] (9.2% of children missed school at least once during one of the test sessions), violation of sphericity, as well as large interindividual variabilities [70] and is routinely used in eye movement studies. To study the difference of digital exploration patterns between good and poor readers, we split our experimental group into two additional subgroups: good and poor decoders according to their decoding skill on the last syllable and letter decoding test using a cutoff at the median level. Thus, the ‘good decoder’ group is composed of good decoders from Group 1 and Group 2, and the same is true for the ‘poor decoder’ group. We used the *lmer* function with decoding skill (high vs. low), Session (from 1 to 6) and Phase (1 vs. 2) as fixed effects and participants as random effects. The model for each dependent variable was:


$$\begin{array}{c} \text{Finger\textbackslash{} movement\textbackslash{} variable}\;\sim \;\text{Decoding\textbackslash{} skill*Session*Phase\textbackslash{} + \textbackslash{} (1|subject) }+\;\in \end{array}$$

Changes in finger movements were also analyzed to assess the effects of word length (defined as a random effect factor) on the motor output. In this analysis, finger movements were analyzed for sentence trials (trials 16–20 of fourth to sixth sessions in each phase). We used the following model:


$$\begin{array}{c} \text{Finger\textbackslash{} movement\textbackslash{} variable}\;\sim \;\text{Decoding\textbackslash{} skill*Session*Phase\textbackslash{} + \textbackslash{} (1|subject)\textbackslash{} + \textbackslash{} (1|word) }+\;\in \end{array}$$

For clarity, all LMM results using Satterthwaite’s method of estimating degrees of freedom. As previously shown, during reading the landing position of eye movement saccades on words depends on the text lexical properties such as world length and is a sensitive index of reading efficiency, as the word length effect is more pronounced in children than in adult readers [44,46,71]. To determine whether digital saccades also demonstrate word length effect (short-1–6 letters- vs. long-7–11 letters) with developing decoding skills, we used the following LMM:


$$\begin{array}{c} \text{Finger\textbackslash{} kinematic\textbackslash{} Variable}\;\sim \;\text{Decoding\textbackslash{} skill*Session*Group}\;+\;(1|\text{subject})\;+\;(1|\text{word})\;+\;\in . \end{array}$$

We considered both the position of the first fixation and the number of fixations and applied this analysis to a subset of trials in which whole sentences were presented to children.

## Results

### 1. Impact of digit-tracking vs paper modality on reading tasks

**Fig 2:**
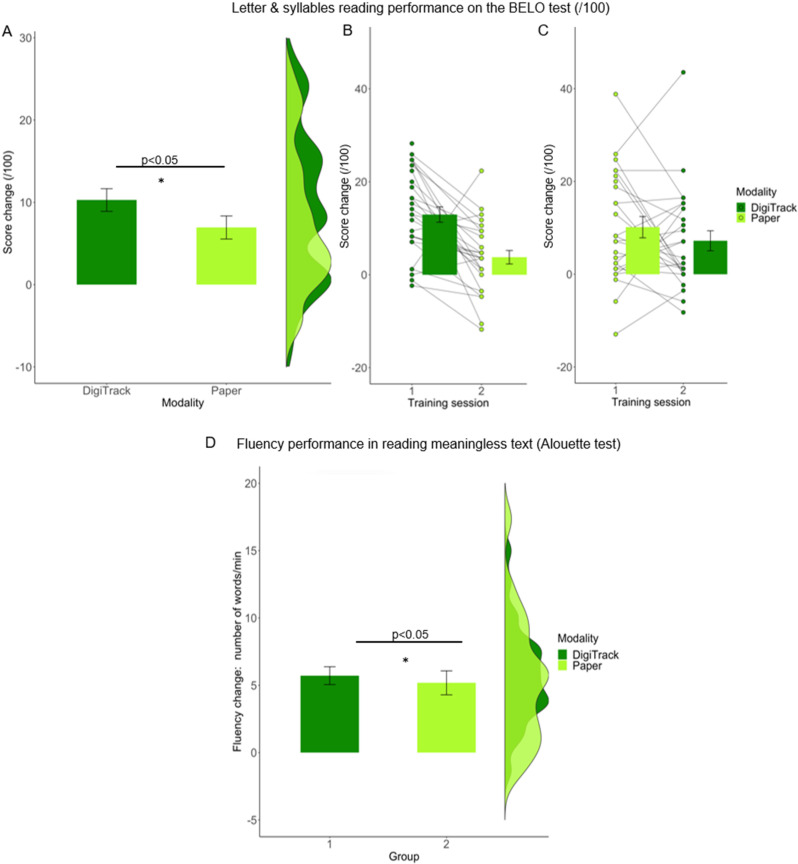
Change in letter and syllable reading (BELO) and fluency performance in reading meaningless text (Alouette test) during digit-tracking vs paper. (A) Boxplot and violin plots show that children in the digit-tracking (dark green) progressed more on letter and syllable reading (+10.28 on performance score) compared to those trained with paper modality (light green). (B) Bar plot and individual data points of Group 1 trained with digit-tracking on Phase 1 (+12.94 scores). (C) Bar plot and individual data points of group 2 trained with Paper on phase 1 (+10.14 scores) and digit-tracking on phase 2 (+7.20 scores). Both in **B** and **C** individuals data points are connected by a line. (D) Fluency performance during reading meaningless text (Alouette test) for phase 1 training. Children trained with the digit-tracking modality (dark green) progressed significantly more (+5.71 words per minute) than those trained with Paper modality (+5.18 words per minute, p = 0.048). Error bars represent the standard error of the mean (s.e.m.).

Results from the LMM on percent change in letter and syllable reading show a significant effect of modality (Fig 2, F (1,30) = 6.32, *p *= 0.02, CI = [0.86, 7.98], R²_model_ = 0.31, R²_modality_ = 0.05): children who were trained with digit-tracking made more progress in letter and syllable reading (+10.28 score, *SE = *1.38) than children trained in the paper modality (+6.95 scores, *SE = *1.40). We also found a significant effect of phase (F(1,30) = 7.05, *p *= 0.01, CI = [1.11, 8.23], R²_phase_ = 0.09): children improved more during the first training phase (+11.61 scores, *SE = *1.39) compared to the second one (+5.44 scores, *SE = *1.29). Additionally, there was a significant effect of age (*b = *−17.72, *SE = *4.71, *p *< 0.001, R²_age_ = 0.05), with younger children showing greater progress than older ones.

A similar analysis was carried out on **meaningless text reading** to evaluate changes in reading fluency and accuracy. The chosen model had the lowest AIC (409.47 compared to a minimal model without covariates: AIC = 603.73). Results from the LMM on reading accuracy showed a significant effect of training phase (F (1,30) = 4.51, *p *= 0.04, CI = [0.20, 8.27], R²_model_ = 0.11, R²_phase_ = 0.03) as reading accuracy improved more in the first (+8.68%, *SE = 1.56*) than the second phase of training (+3.55%, *SE = *1.05). Results from the LMM on reading fluency show a significant interaction effect between modality and phase (F (1,30) = 4.23, *p *= 0.049, R²_model_ = 0.18, R²_modality:phase_ = 0.06): the digit-tracking group improved in reading speed more than the paper group in the first phase (digit-tracking: + 5.71 words per minutes, *SE = *0.66; paper: + 5.18 words per minute, *SE = *0.89) but not in the second phase of the experiment (digit-tracking: + 6.53 words per minute, *SE = *0.74; paper: + 6.94, *SE = *0.91). There was no significant effect of age (*b = *4.19, SD = 2.23, *t *= 1.88, *p *= 0.07), and no effect of motor skills (*b = *−0.351, SD = 0.19, *t *= −1.90, *p *= 0.07).

### 2. Final evaluation in June

**Fig 3:**
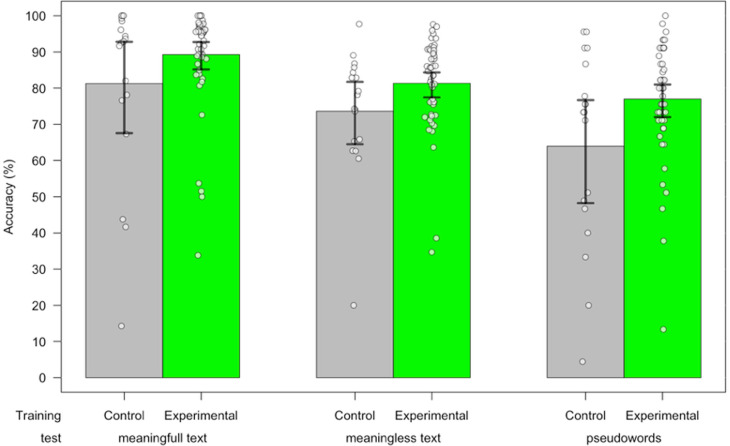
Reading accuracy expressed as percentage of words correctly read, according to the type of text (left, meaningless; middle, meaningful, right, pseudowords) for control (grey) and experimental (green) groups. Error bars represent 95% confidence intervals.

The timeline of our evaluation was presented in S4.Fig in S1 File. We performed an ANOVA on accuracy and fluency (reading speed), with the training group as between-subject factor (Experimental vs Control) and reading tests (pseudowords, meaningless text and meaningful text reading) as within-subject factor. There was a significant training group effect (F (1,67) = 5.36, *p *= 0.02, CI = [−17.81, −1.32]) on reading accuracy: the percentage of average words correctly read (average across all tests) in the experimental group (*M = *82.52, *SE = *1.21) was higher than in the control group (not trained) (*M = *72.95, *SE = *3.25). We also observed an effect of the type of test (F (1,67) = 32.14, *p *< 0.001) on the accuracy but no interaction effect (F (2, 134) = 1.31, *p *= 0.27): in June, children were able to read correctly 73.57% (*SE = *2.42) of pseudowords, and 79.32% (*SE = *1.70) of words from the meaningless text and 87.17% (*SE = *2.10) of words in the meaningful text. There was no group training effect on reading fluency (F (1.67) = 1.59, *p *= 0.21, CI = [−18.12, 4.09]) neither an interaction effect (F (2,134) = 1.24, *p *= 0.29), but a main effect of the type of test (F (2.134) = 139.54, *p *< 0.001): children read pseudowords at a rate of 20.15 per minute (*SE = *1.24), words in meaningless text at a rate of 43.219 per minute (*SE = *2.61) words in meaningful text at a rate of 60.41 per minute (*SE = *3.73). Reading accuracy data are presented in Fig 3.

### 3. Finger kinematics and decoding reading performance

We assessed whether finger movements recorded during digit-tracking exercises provide information about reading performance. By analogy with eye movement analysis, we considered the following finger movement variables: digital fixation duration, proportion of digital regressive saccades, digital saccade length and speed. To examine differences in finger trajectory as a function of reading ability, we divided each experimental group into subgroups of good and poor readers. To do so, we used as a cut-off median scores on syllables and letters decoding test performed after the digit-tracking training phase (post-test 1 for Group 1 and post-test 2 for Group 2) (see S1.Fig in S1 File for the procedure, S5.Fig, S6.Fig, S7.Fig, S3.Text, S4.Text and S5.Text in S1 File for replication with other tests). For each dependent variable, LMM was fit to the data with participants as random effect: Movement variable *~* Decoding skill * Session*Phase + (1|subject) + ∊.

**Fig 4:**
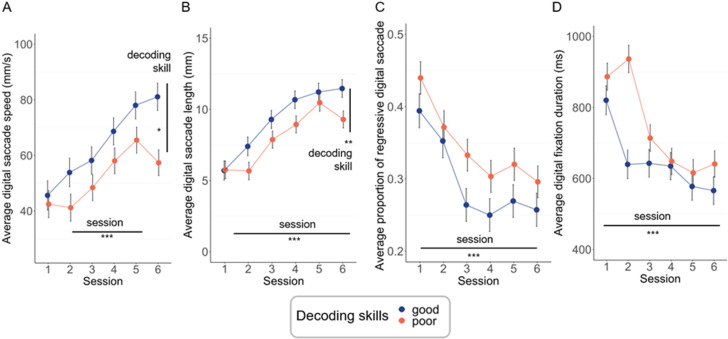
Change in finger movements according to decoding level (blue: good decoders, orange: poor decoders) and session (1 to 6) for Group 1. (A) Average speed of digital saccade (mm/s). (B) Average length of digital saccades (mm). (C) Proportion of digital regressive saccades. (D) Average duration of each digital fixation in milliseconds. Significance levels: *is for p < 0.05; ** is for p < 0.01; *** is for p < 0.001.

Results from the LMM show that finger movements predict the performance achieved by the children at the end of digit-tracking training. Most of these parameters change progressively from session to session within the training phase and these changes are most pronounced for finger data acquired in Phase 1 (Fig 4A–D). Data and figures from Phase 2 are available in S2.Fig in S1 File.

For *digital saccade speed* (See Table 2 for detailed beta and t-values), we found significant effects on decoding skills (F(1, 50) = 7.97, *p *= 0.007, CI = [3.91, 23.23], R²_model_ = 0.025, R²_decoding skills_ < 0.001), phase (F (1,50) = 18.86, *p *< 0.001, CI = [−30.54, −11.22], R²_phase_ = 0.001) and sessions (F (5,90703) = 116.00, *p *< 0.001): digital saccades were faster in good readers, in readers in Phase 2 and increased from the third to the fifth session of training. There was an interaction effect between session and decoding skill (F (5, 90703) = 11.53, *p *< 0.001, and between phase x session x decoding skill effect (F (5, 90703) = 23.83, *p *< 0.001): for phase 1, the higher saccade speed of good decoders was observed at the end of the training, whereas at phase 2, this gap was highest at the fourth and fifth sessions of phase 2.

**Table 2:** Effects obtained by LMM for the contrasts of interest and for each finger movement variable. Significant effects (|t| > 1.96) are in bold.

	Digital saccade speed			Digital saccade length			Proportion of regressive saccades			Fixation duration		
	**b**	**SE**	**t**	**b**	**SE**	**t**	**b**	**SE**	**t**	**b**	**SE**	**t**
Phase1 - Phase2	**−20.88**	**4.81**	**−4.34**	**−2.48**	**0.62**	**−4.01**	−0.01	0.02	−0.3	**175.13**	**38.16**	**4.59**
session1 - session2	−2.15	1.43	−1.5	**−0.38**	**0.18**	**−2.13**	**0.04**	**0.01**	**4.35**	**39.52**	**10.5**	**3.76**
session2 - session3	−2.38	1.24	−1.91	**−1.44**	**0.16**	**−9.25**	**0.06**	**0.01**	**6.47**	**42.63**	**9.64**	**4.42**
session3 - session4	**−9.33**	**1.01**	**−9.21**	**−1.41**	**0.13**	**−11.18**	**0.04**	**0.01**	**4.16**	**20.57**	**8.39**	**2.45**
session4 - session5	**−6.15**	**0.96**	**−6.44**	**−0.71**	**0.12**	**−5.93**	−0.01	0.01	−0.63	**43.66**	**8.08**	**5.41**
session5 - session6	0.83	0.97	0.86	0.05	0.12	0.43	0.00	0.01	−0.23	0.1	8.2	0.01
good – poor	**13.57**	**4.81**	**2.82**	**1.5**	**0.62**	**2.42**	−0.03	0.02	−1.4	−68.6	38.16	−1.8
Phase1:session1:good - poor	3.16	7.17	0.44	−0.05	0.92	−0.05	−0.06	0.03	−1.85	−66.64	55.98	−1.19
Phase1:session2:good - poor	12.67	7.08	1.79	1.73	0.91	1.9	−0.02	0.03	−0.61	**−296.93**	**55.85**	**−5.32**
Phase1:session3:good - poor	9.77	6.79	1.44	1.41	0.87	1.62	**−0.08**	**0.03**	**−2.36**	−71.14	54.15	−1.31
Phase1:session4:good - poor	10.61	6.73	1.58	**1.74**	**0.86**	**2.01**	**−0.07**	**0.03**	**−2.07**	−13.36	53.73	−0.25
Phase1:session5:good - poor	12.53	6.75	1.86	0.75	0.87	0.86	−0.07	0.03	−1.94	−38.46	53.93	−0.71
Phase1:session6:good - poor	**23.74**	**6.77**	**3.5**	**2.18**	**0.87**	**2.5**	−0.05	0.03	−1.61	−74.95	54.11	−1.39
Phase2:session1:good - poor	**15.93**	**7.53**	**2.12**	1.88	0.97	1.94	0.01	0.04	0.3	−58.47	59.00	−0.99
Phase2:session2:good - poor	3.79	7.48	0.51	0.13	0.96	0.14	−0.01	0.04	−0.41	14.24	58.83	0.24
Phase2:session3:good - poor	8.75	7.34	1.19	1.3	0.94	1.38	0.00	0.04	0.13	−81.52	58.33	−1.4
Phase2:session4:good - poor	**31.54**	**7.25**	**4.35**	**3.8**	**0.93**	**4.07**	0.03	0.04	0.81	−67.56	57.77	−1.17
Phase2:session5:good - poor	**22.99**	**7.26**	**3.16**	**2.09**	**0.93**	**2.24**	−0.02	0.04	−0.49	−43.12	57.84	−0.75
Phase2:session6:good - poor	7.37	7.24	1.02	1.06	0.93	1.14	−0.03	0.04	−0.84	−25.24	57.67	−0.44

For *the digital saccade length* (See Table 2 for detailed beta and t-values), the analysis showed significant effects of decoding skill (F(1, 50) = 5.87, *p *= 0.02, CI = [0.26, 2.74], R²_model_ = 0.03, R²_decoding skill_ < 0.001), phase (F (1, 50) = 16.08, *p *< 0.001, CI = [−0.73, −0.03], R²_phase_ = 0.002) and sessions (F (5, 90702) = 261.99, *p *< 0.001): digital saccade length were longer in good readers (good-poor: *b = *1.50, *SE = *0.62, *t *= 2.42, *p *= 0.02), in readers from Phase 2 and increased from the first *t*o the fifth session of training. The interaction of phase x session effect was significant (F(5,90702) = 14.48, *p *< 0.001): digital saccade length increased more in Phase 1 (session 1: mean = 5.73 mm, *SE = *0.46; session 6: mean = 10.38 mm, *SE = *0.44) than in Phase 2 (session 1: mean = 9.63 mm, *SE = *0.48; session 6: mean = 12.76 mm, *SE = *0.47), again due to the greater margin of progression of children trained early in the school year. Session x decoding skill interaction effect was significant (F (5,90702) = 13.16; *p *< 0.001): and a phase x session x decoding skill interaction effect was also found (F (5, 99681) = 4.94, *p *< 0.001): the difference between good and poor decoders was relatively stable at training phase 1 (group 1) but had important fluctuations at training phase 2. The analysis on the *proportion of digital regressive saccades*, showed no effects of phase and decoding skill (*ps > *0.32). There was a significant effect of session (Fig 4B, F (5, 8293) =77.81, *p *< 0.001): the proportion of regressive saccade decreased from the first to the fourth session (session 1–2: *b = *0.04, *SE = *0.01, *t *= 4.35, *p *< 0.001, CI = [0.02, 0.06]; session 2–3: *b = *0.06, *SE = *0.01, *t *= 6.47, *p *< 0.001, CI = [0.04, 0.08]; session 3–4: *b = *0.04, *SE = *0.01, *t *= 4.16, *p *< 0.001, CI = [0.02, 0.06]; all |*t*|s < 0.63 for other sessions).

The analysis on *duration of digital fixation* showed no main effects of decoding skill (F (1, 50) = 3.23, *p *= 0.08, CI = [−145.25, 8.06], R²_model_ = 0.013). We found a significant phase effect (F (1, 50) = 21.06, *p *< 0.001, CI = [98.47, 251.79], R²_phase_ = 0.001): the duration of digital fixations was longer in phase 1 compared to phase 2. We also found a triple interaction between phase x sessions x decoding skill (F (5, 144905) = 22.24, *p *< 0.001): the gap between good versus bad decoders in Phase 1 is greater than in Phase 2 (See S2.Fig) in S1 File, especially in the second session. Finally, we observed a significant effect of session (within the training phase) (F (5, 209579) = 76.00, *p *< 0.001): children reduced their digital fixation durations from the first to the fifth session and this effect was stronger in Phase 1 than in Phase 2: (phase x session interaction: F (5, 144905) = 48.20, *p *< 0.001). It was also stronger in poor decoder than in bad decoder (decoding skill x sessions (F (5, 144905) = 22.24, *p *< 0.0001).

**Fig 5:**
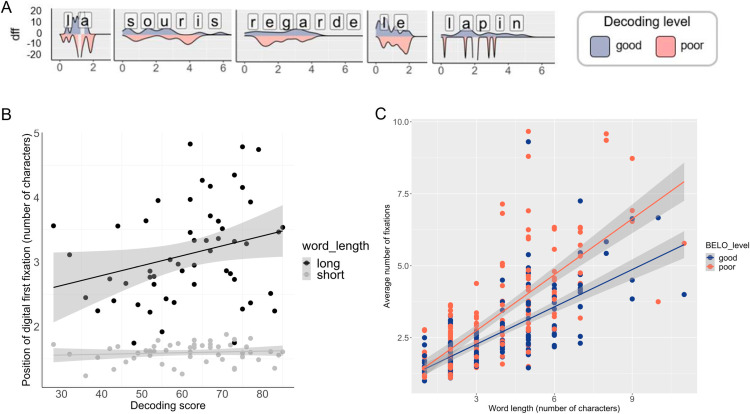
Decoding motor patterns as a function of word length. (A) Smoothed frequency of first fixation (dff) position according to the position in the word (relative position in characters, characters are overlaid with the graph for representational purpose, the first letter starts in the position 0 and end in the position 1 and so on) for good decoders (in blue, upper part of the line) and poor decoders (in orange, lower part) for each word of the 5-words sentence “The mouse looks at the rabbit” in French: “La souris regarde le lapin”). The frequency refers to the number of children in each group whose first fixation fell into a given segment of the word and frequency histograms were smoothed into Gaussian curves using algorithm describe in Materials & Methods section. We can observe that in long words (like “regarde”), the peak of density for poor decoders didn’t fall toward the center of the word but over the first two letters, whereas for good decoders the peak fell between the fourth and the fifth letter. (B) Position of the first digital fixation on the word (expressed in number of characters) according to children’s decoding score using the BELO test (47), and for word length. The position of the first digital fixation depends on the word length: it occurs around the 1st letter in short words (in light grey), and around the 3rd letter in long words (dark grey). Word length effect is stronger in good decoders than in poor decoders. (C) Average number of fixations in good (blue dots) and poor decoders (red dots) as a function of word length (expressed in number of characters) in both training sessions. Regression lines show the slope for good (in blue) or poor (in red) decoders. The number of fixations increased with word length. This effect was stronger in poor decoders than in good decoders. Here only two examples of word were shown (“lapin” and “regarde”).

Results from the LMM on the position of the first digital fixation show a significant effect of word length (short-long: *b = *−1.46, *SE = *0.17, *t *= −8.40, *p *< 0.001, R²_model_ = 0.10, R²_word length_ = 0.001): the position (in number of characters) of the first digital fixation occurs around the 1^st^ letter in short words (mean = 1.60, *SE = *0.02), and around the 3^rd^ letter in long words (mean = 3.14, *SE = *0.09). There was a significant effect of decoding skills (poor-good: *b = *−0.42, *SE = *0.10, *t *= −3.99, *p *< 0.001); poor decoders made *t*heir first digital fixation more toward the beginning of the word than good decoders (Good decoders: mean = 1.83, *SE = *0.03; poor decoders: mean = 1.74, *SE = *0.03). One dimensional density map built from all individual from each group (good *vs.* poor decoders) and overlaid on words from sentences shows that in children with poor decoding skills, for long words such as “*regarde*”, fixations occurs mainly at the beginning of the word, but at near the center of the word for good decoders (Fig 5A). There was a significant interaction effect between word length and decoding score. The effect of word length was stronger in good decoders than in poor decoders (short-long x poor-good: *b = *0.37, *SE = *0.11, *t *= 3.47, *p *< 0.001, R²_decoding skills:word length_ = 0.002) (Fig 5B).

Finally, the analysis on the number of digital fixations showed a robust effect of word length (F (1, 70.25) = 92.23, *p *< 0.001, R²_word length_ = 0.023): the number of fixations increased linearly with word length (in number of characters). There was no effect of decoding skills but a significant interaction between word length and decoding skill (F (1, 3805.63) = 31.60, *p *< 0.001, R²_decoding skills:word length_ = 0.006): the effect of word length was stronger in poor decoders than in good decoders (See Fig 5C)).

### 4. Finger kinematics and reading fluency at the end of the school year

Based on children’ performances on standard linguistic tests (fluency of pseudowords, meaningless and meaningful text reading) as assessed at the end of the year (see Methods & S1.Text in S1 File for description of tests), participants were divided into good or poor readers within each experimental group (group 1 vs group 2) using the median value as a cut-off. We analyzed the same kinematics variables presented in Fig 3 (*digital saccade speed*, *digital saccade length*, *proportion of digital regressive saccades* and *digital fixation duration*). Significant main effect of all reading levels were observed for *saccade speed* (Pseudowords level: F (1,46) = 12.34, *p *= 0.001, good – *p*oor: *b = *16.83, *SE = *4.79, *t *= 3.51, *p *= 0.001, CI = [7.19, 26.47], R²_model_ = 0.03, R²_reading level_ < 0.001; Meaningless text level: F (1,46) = 15.50, *p *< 0.001, good – *p*oor: *b = *18.19, *SE = *4.62, *t *= 3.94, *p *< 0.001, CI = [8.89, 27.48], R²_model_ = 0.04, R²_reading level_ < 0.001; Meaningful tex*t* level: F (1,46) = 5.78, *p *= 0.02, good – *p*oor: *b = *12.37, *SE = *5.14, *t *= 2.40, *p *= 0.02, CI = [2.01, 22.72], R²_model_ = 0.03, R²_reading level_ < 0.001) and *saccade length* (Pseudowords level: F (1,46) = 8.50, *p *= 0.005, good – *p*oor: *b = *1.87, *SE = *0.64, *t *= 2.92; *p *= 0.005, CI = [0.58, 3.16], R²_model_ = 0.04, R²_reading level_ < 0.001; Meaningless tex*t* level: F (1,46) = 15.50, *p *< 0.001, good – *p*oor: *b = *2.15, *SE = *0.62, *t *= 3.46, *p *= 0.001, CI = [0.90, 3.39], R²_model_ = 0.04, R²_reading level_ < 0.001; Meaningful tex*t* level: F (1,46) = 5.78, *p *= 0.02, good – *p*oor: *b = *1.45, *SE = *0.68, *t *= 2.14, *p *= 0.04, CI = [0.85, 2.81], R²_model_ = 0.03, R²_reading level_ < 0.001): good readers made longer and faster digital saccades com*p*aring to poor readers. Main effec*t* of Meaningless text level was observed for *fixation duration* (F (1,46) = 5.60, *p *= 0.02, R²_model_ = 0.02, R²_reading level_ < 0.001): good readers made shorter fixations than *p*oor readers (good – poor: *b = *−89.74, *SE = *37.94, *t *= −2.37, *p *= 0.02, CI = [−166.12, −13.36]) (A full descri*p*tion of statistical results are reported in S3.Text, S4.Text, and S5.Text) in S1 File.

## Discussion

In this study, we show that when learning to read, training children on a tablet screen where parafoveal information is dynamically masked using a blur and unmasked by finger pointing facilitates reading acquisition compared to conventional paper-based training without masking. We demonstrate this effect in first graders using a training program based on a randomized controlled cross-over design. Moreover, we provide evidence that the pattern of digital exploration observed with the finer movement tracking method reflects reading accuracy, its evolution with experience and could also be used to predict the readers’ initial skills.

Our goal was twofold. First, to improve reading acquisition, we tested the efficacy of removing parafoveal orthographic interference and finger pointing attentional cueing using the digit-tracking method. Second, we assessed whether finger movements data acquired during digital text exploration yielded a pattern similar to that obtained with direct eye movement recordings.

Our results show that children trained in reading exercises with digit-tracking improved their decoding ability (reading letters, phonemes and syllables) and, during the first training, the number of correctly read words per minute significantly increased comparing to when they were trained in the same exercise presented on paper. Our interpretation is that our digit-tracking method increases attention to word units (letters, syllables) and facilitates their identification, making it easier to read word and sentences. Therefore, monitoring parafoveal crowding and using finger text scanning can promote selective attention to foveal word information. Indeed, to become a proficient reader letter and word identification must be processed automatically in order to efficiently allocate attentional resources to sentence comprehension [72,73]. By blurring the parafoveal visual field and pointing to specific letter and syllables, it could help strengthen the identification of letters and syllables and improve children’s decoding skills, which is a key component of long-term reading abilities.

Hence, digit-tracking is a tool that helps children to overcome the barrier of crowding at the very start of their learning path, making it possible to memorize and combine the small building blocks of reading, and gradually become an independent reader. As previous studies have shown, children with good decoding skills are likely to have greater benefit from previewing which is expected to enhance reading fluency [33]. Also, in early reading acquisition, reading comprehension is linked to performance on decoding capacities [74]. Therefore, we can argue that, training with the digit-tracking modality it strengthens decoding skills in the early stages of reading acquisition which in turn improves reading fluency. Indeed, we found an early effect of training modality: children made better progress in letter and syllable reading after only four weeks of training with the digit-tracking method. This effect is observed with only 3 hours per week of training spread over 6 weeks, which is a very short exposure, as teachers typically spend 10 hours per week on reading acquisition in first-grade [75].

Overall, our results are consistent with other findings obtained with crowding-free modalities such as Word Mode [31] or increasing inter-letter spacing [28,30] that have shown a positive effect on reading. Another study has degraded visually parafoveal stimuli, *i.e.,* to exchange randomly black and white pixels of letters [33] to reduce parafoveal costs. However, these methods face criticisms as they still induce attentional costs [76]. The novelty of our approach is to gradually mask parafoveal stimuli using a gaussian blur, thus making visual information far from the fixation point temporarily unavailable. Overall, our results are also consistent with other findings obtained with multimodal processing of letters and pointing employed to prime children’s attention to printed text [7,8,10,77]. The pointing gesture required to unblur the text items further contributes to attentional cueing, an approach that has been shown to reduce the cost of crowding [34].

In our study, we investigated how the digit-tracking method could enhance children’s decoding and fluency skills, two essential components of word reading automatization, and reading proficiency [78]. Indeed, reading difficulties are explained by deficits in phonological processing [79–81], rapid automatized naming [82] and/or visual attentional processing [57], all of which directly impact decoding and fluency. While text comprehension is undeniably important for reading [83], it depends on higher-order cognitive processing of words and sentences, and is not exclusive to reading.

Within the theory of automatic information processing [72], automatizing word identification allows for the reallocation of attention to text comprehension, highlighting the importance of strengthening reading-specific skills, such as letter and grapheme decoding. In beginners, visual processing of letter and word identification can be disrupted by visual crowding [84]. Our intervention specifically aims to reduce crowding to enhance letter and word recognition to improve reading performance. Therefore, decoding and fluency, key indicators of reading improvements, are the most relevant measures for assessing the effectiveness of our method.

Our study focuses on decoding and fluency, but we recognize the need to include reading comprehension and real-world tasks in future research for a more comprehensive evaluation. Studies suggest that fluency and comprehension are linked, with progress in one area benefiting the other [85]. Early improvements in word fluency also predict later progress in word and text reading, showing the value of early interventions [86]. Decoding is one of the first skill to be reinforced in beginners [27] in order to facilitate fast reading acquisition. As shown here, crowding can interfere with learning. Hence, we suggest it is important to examine first how to reduce crowding to enhance decoding. In sum, future longitudinal studies should explore the timeline of reading improvements, grounded in evidence of the short-term impact of basic decoding and fluency skills.

A concern may arise which is the possible negative effect resulting from the continuous guidance provided by the digit-tracking technique on later unassisted reading context. The question then is whether, by making the reading task easy (i.e., by eliminating interfering elements and thus reducing the related cognitive load), our method may reduce the readers’ abilities to handle competing information when facing natural reading conditions without assistance. The results of this study do not support this concern as all pre- and post-training reading assessments, as well as the end of school year evaluation, were conducted on paper. Overall, the observed difference between the control (the group not trained) and the experimental groups in the percentage of pseudowords correctly read in the June evaluation session provide some evidence that the trainings (paper + digit-tracking together) were more efficient than “a standard reading school training”, where government reading programs are executed in the classroom by an experienced teacher. Moreover, this also suggests that effects (from the trainings in general not just digit tracking) may last at least in the medium-term (i.e., 4-months after the end of trainings). On the other hand, digit-tracking training resulted in an immediate positive effect on the progression of decoding skills (assessed by the BELO test).

We collected finger movement during the training exercises using pseudowords and sentences stimuli. We were able to show that digital exploration profiles differ among children according to their decoding reading performance as assessed by the BELO test [55] on several measures such as digital saccade length, fixation duration, saccade speed and the position of first fixation. Good decoders have shorter digital fixations as well as longer and faster digital saccades than poor decoders. Interestingly, the overall trend for the finger movement pattern observed for these two groups of decoders (poor and good decoders) were similar when their reading abilities were classified based on other reading tests (pseudowords, meaningless and meaningful text reading fluency) assessed later at the end of the year (See S3.Text, S4.Text, S5.Text and S5.Fig, S6.Fig, S7.Fig in S1 File). These results suggest that digit-tracking could be used to predict reading level and to identify children with reading difficulties at an early stage. To do so, future investigations are needed in order to collect relevant finger movement features on a large number of children throughout their reading development, and to provide standardization and validation of finger movement measures as a screening tool.

Interestingly, the above findings are consistent with previous studies reporting differences between good and poor readers using eye movement measures [24,40,87,88]. For instance, we observed that in good decoders the first fixation lands at a position closer to the center of the word (in long words) than poor decoders, and this was also reported using direct eye movement recording [44]. We also observed effects of word length similar to those observed with the eye tracking method [47], namely that for long words, the first fixation position of good decoders is closer to the center of the word, corresponding to efficient text processing, compared to poor readers for whom the first fixation position is at the beginning of the word [43]. The hypothesis that the finger movements pattern observed in this study in good and poor decoders might reflect a difference in fine motor control or on the level of eye-hand coordination skills is in our view unlikely, given the similarity of reading patterns observed with digit-tracking and eye tracking methods and their modulation by linguistic parameters. Although, these factors may contribute to develop reading skills, we show that linguistic determinants as those observed in eye-movements are also critical. Altogether our results and the findings of Lio et al. suggest that digital exploration is a robust proxy for eye movements scanning to inform cognitive processing during reading.

Little is known about how learning to read during the first school year affects eye movements. Finger movement and fixation patterns recorded with our reading method thus offer a unique window into the initial stages of reading acquisition. We showed a robust effect of training sessions, with a progressive decrease in digital fixation duration, and an increase in saccade length and speed. As children were in their early reading acquisition process, tracking how these variable change over time provides a sensitive indicator of progression in acquisition of basic reading skills such as decoding and reading fluency, and could be used for early detection of reading difficulties and remediation. For instance, visual attentional abilities predict future reading skills [89], and there are findings of dyslexic children with deficits in visual attention span despite normal phonological skills [90,91]. These children were shown to make more fixations indicating a sequential coding of the written words rather than an ability to recognize them quickly at first glance.

The future use of the digital method described here and designed to accelerate decoding and reading fluency needs to be evaluated in the context of large classrooms directly used by teachers or in a familiar environment by parents. It should be noted that in the small-group context of our investigation, children quickly became familiar with the method as most used it without assistance after a single demonstration.

Our study presents some limitations. The cross-over design we chose allowed to overcome interindividual variability (we can compare the modalities within a same child) but only tested the immediate modality effects (by the end all children were trained with both digit-tracking and paper). Our data showed that both trained groups of children (group 1 & 2) obtained higher reading score compared to those (control group not trained) who did not. However, a firm conclusion cannot be drawn given the lack of pre-testing data on the untrained control group and group randomization.

Given that the study included two training phases, one might also question whether a spillover effect occurred—specifically, whether performance in the first phase influenced performance in the second phase. More precisely, training with digit-tracking in the first phase (Group 1) could have either positively or negatively impacted reading progression in the second phase, when these same children were trained on paper. A negative spillover effect would hinder the second phase progression of children from Group 1 in the paper modality, potentially overestimating the benefit of digit-tracking. Conversely, a positive spillover effect would lead to an underestimation of the true effect size of the digit-tracking benefit on reading. To rule out the influence of any potential spillover effect, we can re-examine the conclusions by focusing solely on the first phase of the experiment. This analysis confirms that digit-tracking training improved reading fluency, as Group 1 (trained with digit-tracking) showed greater progress compared to the group trained on paper in the first phase. Furthermore, because all conclusions drawn from finger kinematics are based on the first phase of the experiment, they are unaffected by any spillover effect. The conclusions from the letter and syllables decoding is only significant when considering also the second phase, potentially overestimating the benefit of digit-tracking on this outcome measure.

Moreover, it is legitimate to consider the role of the digital tablet used in our experiments and the excitement its use may have triggered in the children. If we believe that our crowding-free texts and gesture pointing are the main factors to learning to read, we can’t rule out the possibility that the tablet also had a positive effect.

A preliminary pilot experiment specifically testing the effect of the tablet in another group of children supports our arguments. In this study we found no difference in a set of reading tasks (recognizing letters, phonemes, syllables, sound of syllables and words) between children trained on the paper modality and those trained with the tablet modality without digit-tracking (See Materials and Methods and S2.Table in S1 File). Moreover, it must be stressed that the incentive for learning induced by technological devices is related to gamification features (reward and unlock principles – [92]), which were not present in our digit-tracking design. Additionally, recent data shows increased exposition of children to digital devices at very young age [93], and all schools involved in our project offer digital whiteboards and computers in their classrooms, thus making unlikely that the use of the tablet could have a significant motivational role in enhancing learning to read with digit-tracking, compared to paper training.

We acknowledged that these observations do not exclude in a definitive way the potential effect of the digital device and the finger pointing gesture alone, however, from an educational point, the combination of the positive effects of all these features are relevant. In addition to the crowding reduction effect, the interactive nature of the tool should help teachers to manage individual instructions and feedbacks effectively. Also, the data collected from the finger pointing gesture, once validated and standardized, could be useful to teachers and clinicians as a screening tool, as well as to researchers interested in understanding the visual mechanisms of learning to read.

### Broader implications for future research

While the primary focus of this study was on integrating digit-tracking with reading tasks in first-graders, the findings open up potential avenues for future research into sensorimotor learning and its role in reading acquisition. Although our results are specific to young children learning to read, the method could have broader implications beyond this population.

The digit-tracking method we employed may offer promise for future interventions aimed at children with reading disabilities, such as dyslexia, or other cognitive impairments. By tracking motor behaviors (e.g., finger movements) as an indicator of attentional focus [27], this approach could potentially provide educators and clinicians with a tool to support children who face challenges with traditional reading methods.

Given the increasing use of digital tools in educational settings, the tablet-based digit-tracking method is highly relevant to modern pedagogical practices. As digital classrooms become more common, further studies could investigate how educational applications incorporating motor interaction, such as finger-tracking, might enhance reading acquisition. Such tools could also provide real-time feedback to guide learners and teachers through reading tasks adapted to the learner’s ability and help reduce visual crowding, offering potential benefits for learners who struggle with crowded environment. Such methods could be particularly valuable in classrooms where individualized feedback might be challenging to provide on a regular basis, thus helping teachers monitor student progress more effectively.

The use of tablet-based digital assistance might raise concerns about excessive screen time in young children. The association of screen time and cognitive development is an open question, with studies showing negative effects on some measures, and null or positive effects on others [94]. This association also depends on the type of screen activity [95]. A recent meta-analysis showed that higher language skill in children was associated with less screen time and higher quality of screen activity [96]. Here, we use a short-term intervention (6 sessions of 35 minutes over 4 weeks) targeting specific reading skills, which is in line with recommendations of limited screen time to ensure better quality of screen use. We believe that limiting the use of digit-tracking to first grade and employing it as a complementary educational tool can provide meaningful support in strengthening specific reading skills.

In addition to classroom use, the potential application of digit-tracking methods in home learning environments is equally significant, particularly as digital learning continues to expand beyond formal educational settings. Parents and caregivers could use tablet-based educational tools to support children’s reading development at home. These tools could provide personalized auditory feedback related to the child’s focus of attention, fostering independent learning of grapheme-phoneme association. For example, our team has proposed incorporating features such as auditory cues, where a sound is played when a child successfully unblurs a specific portion of the text. This type of interaction could make learning more engaging and interactive. Such tools could be especially valuable for children who need additional support outside of school hours, offering an accessible way to reinforce learning in a stimulating and user-friendly format.

Furthermore, cross-linguistic research has revealed that cognitive precursors of reading, such as phonological awareness and decoding skills, can vary across languages due to differences in orthographic depth and linguistic structures. For instance, Landerl and colleagues [85] examined these precursors in different orthographies, emphasizing the need to consider language-specific factors in reading interventions. Therefore, future research should explore the applicability of our digit-tracking method across different contexts to determine its generalizability and effectiveness in various languages.

This study suggests that digit-tracking may enhance early reading skills in French children. If the long-term positive effects our method are validated across languages, its scalability and equity warrant further consideration. While beyond this study’s scope, prior research has examined the scalability of digital tools. For example, the ABRACADABRA literacy program in Kenya expanded from 12 to over 500 classrooms due to stakeholder engagement, local adaptability, and continuous evaluation [97]. Regarding equity, tablet costs pose a challenge in low-income countries, but initiatives like Rumie [98] have successfully provided affordable, preloaded tablets in over 20 countries, improving access to education. Future studies should indeed address these aspects to ensure that the digit-tracking method is accessible, equitable, and appropriately balanced within educational contexts.

Finally, future research could extend this line of inquiry to explore the use of digit-tracking in other cognitive domains, such as mathematical problem solving, geometry, or even subjects like history or biology. Investigating its potential in fostering sensorimotor learning and reducing crowding effects across a variety of educational tasks, both in classrooms and home environments, could significantly broaden the utility of this approach.

## Conclusion

In summary, we have shown that the removal of parafoveal crowding with the digit-tracking method on a tablet screen combined with a finger pointing gesture, has a beneficial effect on the decoding of written information and progression of reading performance. No negative neither positive effect purely related to the presence of a digital device (Hawthorne effect) was detected. Besides offering a crowding-free text environment and strengthening attentional focus by finger pointing gesture, this method could also be a potential tool for detecting early reading difficulties, as digital exploration patterns recorded were informative and consistent with results of eye movement studies. In conclusion, we present a reading method that can accelerate children’s decoding abilities in the first phase of learning to read, that is also useful to teachers for managing individual learning in the classroom thanks to its interactivity functions, and to clinicians and researchers due to the informative nature of data collected on finger movements.

## Supporting information

S1 File **S1.Text**. Testing materials. **S2.Text.** Comparison of the experimental group (Group 1 & Group 2) vs. control group (children in the same class as the experimental group but who did not participate in the training sessions). The active control group was trained with the classical school curriculum by their teacher (n = 4 classes). Teacher participation was voluntary. The 4 teachers who participated were equally experienced (mid-to-late stages of careers). All children were assessed by teachers on the French National reading evaluations, in September and January, and by the research team on reading evaluations (pseudoword reading and meaningful text reading) in June. Only one teacher agreed to share the data from his class on the national evaluation (n = 24, 8 in the control group and 16 in the experimental group).**S3.Text**. Digital saccade as a function of fluency level during pseudowords reading. **S4.Text**. Digital saccades as a function of reading fluency performance for meaningless text (Alouette). S5.Text. Finger movement as a function of reading fluency performance for meaningful text (Monsieur Petit). **S1.Table**. Training stimuli. Overall structure and choice of pseudowords stimuli. In each phase, 20 monosyllabic, 60 bisyllabic and 40 trisyllabic pseudowords were introduced. The complexity of each pseudowords ranged from 1) simple graphemes-simple syllables (CV structure) to 2) complex graphemes-simple syllables (CV structure) and finally 3) complex syllables (CVC or CCV structure). **S2.Table**. Reading results of control study. Mean and SD for each reading-related skill and each modality (Paper or tablet) were presented, along with p-value of two-sample t-test. **S1.Fig**. Data of finger kinematics were collected during 6 training sessions (at phase 1 for Group 1 and phase 2 for Group 2). Within each group, children were divided into good or bad decoders based on their decoding score using BELO test. **S2.Fig**. Change in digital saccade variables according to text decoding level (good vs. poor decoders) and session (1–6) for Group 2. A. Average speed of digital saccade (mm/s). B. Average length of digital saccades (mm). C. Proportion of digital regressive saccades. D. Average duration of each digital fixation in milliseconds. Error bars represent standard error estimated by LMM. Significance levels:. 0.1 < p < 0.05; * 0.05 < p < 0.01; ** 0.01 < p < 0.001, *** p < 0.001. **S3.Fig**. Example of syllable recognition test. Children had to circle the syllable ‘VI’ among six elements proposed underneath. **S4.Fig**. Procedure with a control group included. Along with 54 children of the experimental group, a control group of 19 children from the same classes but did not participate in training were tested in June by our research team. Furthermore, a subset of data from the two national evaluations (in September and January) were collected. **S5.Fig**. Change in finger movement according to pseudowords reading fluency level (good vs. poor readers) and session (1–6) for Group 1. A. Average speed of digital saccade (mm/s). B. Average length of digital saccades (mm). C. Proportion of digital regressive saccades. D. Average duration of each digital fixation in milliseconds. Error bars represent standard error estimated by LMM. Significance levels:.0.1 < p < 0.05; * 0.05 < p < 0.01; ** 0.01 < p < 0.001, *** p < 0.001. **S6.Fig**. Change in finger movement according to reading fluency level for meaningless text (good vs. poor readers) and session (1–6) for Group 1. A. Average speed of digital saccade (mm/s). B. Average length of digital saccades (mm). C. Proportion of digital regressive saccades. D. Average duration of each digital fixation in milliseconds. Error bars represent standard error estimated by LMM. Significance levels:.0.1 < p < 0.05; * 0.05 < p < 0.01; ** 0.01 < p < 0.001, *** p < 0.001. **S7.Fig**. Change in finger touch variables according to meaningful text reading fluency level (good vs. poor readers) and session (1–6) for Group 1. A. Average speed of digital saccade (mm/s). B. Average length of digital saccades (mm). C. Proportion of digital regressive saccades. D. Average duration of each digital fixation in milliseconds. Error bars represent standard error estimated by LMM. Significance levels:. 0.1 < p < 0.05; * 0.05 < p < 0.01; ** 0.01 < p < 0.001, *** p < 0.001
(ZIP)
