# Supplementary material for: A crowding free digital interface to help French-speaking children learn to read
Source: PLoS One. 2025 Jun 25;20(6):e0323623. doi: 10.1371/journal.pone.0323623 (PMC12193705; doi:10.1371/journal.pone.0323623)
Supplement: S1 File — S1.Text. Testing materials. S2.Text. Comparison of the experimental group (Group 1 & Group 2) vs. control group (children in the same class as the experimental group but who did not participate in the training sessions). The active control group was trained with the classical school curriculum by their teacher (n = 4 classes). Teacher participation was voluntary. The 4 teachers who participated were equally experienced (mid-to-late stages of careers). All children were assessed by teachers on the French National reading evaluations, in September and January, and by the research team on reading evaluations (pseudoword reading and meaningful text reading) in June. Only one teacher agreed to share the data from his class on the national evaluation (n = 24, 8 in the control group and 16 in the experimental group).S3.Text. Digital saccade as a function of fluency level during pseudowords reading. S4.Text. Digital saccades as a function of reading fluency performance for meaningless text (Alouette). S5.Text. Finger movement as a function of reading fluency performance for meaningful text (Monsieur Petit). S1.Table. Training stimuli. Overall structure and choice of pseudowords stimuli. In each phase, 20 monosyllabic, 60 bisyllabic and 40 trisyllabic pseudowords were introduced. The complexity of each pseudowords ranged from 1) simple graphemes-simple syllables (CV structure) to 2) complex graphemes-simple syllables (CV structure) and finally 3) complex syllables (CVC or CCV structure). S2.Table. Reading results of control study. Mean and SD for each reading-related skill and each modality (Paper or tablet) were presented, along with p-value of two-sample t-test. S1.Fig. Data of finger kinematics were collected during 6 training sessions (at phase 1 for Group 1 and phase 2 for Group 2). Within each group, children were divided into good or bad decoders based on their decoding score using BELO test. S2.Fig. Change in digital saccade variables according to text decoding level [file pone.0323623.s001.zip › Supplementary_info/S1.Text.docx]

## Testing materials

### Evaluation of letters and syllables naming: BELO ®

The BELO (Batterie d'évaluation de la lecture et de l'orthographe, (63)) is a French standardized test for evaluating reading and orthography. We selected a decoding subtest where children had to read aloud a set of 85 orthographic elements from a printed booklet:

- 26 letters (simple graphemes, e.g. l, p, a)
- 26 simple syllables (11 Consonant-Vowel simples, e.g. da, mi, ul ; 15 Consonant-Vowel-Consonant, e.g. fur, par, gor)
- 33 complexes syllables (including complex graphemes, e.g. ph, euil, oin).

If the child gave 5 wrong answers in a row, the test was stopped. The total score for each child is the sum of elements correctly read (1 point per element read) with a maximum of 85 points.

We computed the BELO score before and after each training phase (4 times in total). For each child and each phase, we computed the change of correctly read elements as the difference between the number of elements correctly read before and after each training divided by the maximum (85) and multiplied by 100.

### Evaluation of meaningless text reading: Alouette test (Alouette R, (66))

The Alouette test is French 265-word text that children read aloud within a 3-minutes time limit. This test is widely used to assess reading skills for children aged between 5 to 14 years-old. Sentences can be meaningless but remain grammatically and structurally correct. The text is presented in such a way that readers are prone to contextual errors: a drawing of a squirrel - *écureuil* in French - is located near the word *écueil* - meaning pitfall; the word *poison* (meaning poison), whose orthographic form is close to *poisson* (fish), is located just after *lac* (lake); familiar expression modified ("Au clair de la lune" became "Au clair de lune"); words with irregular orthography or silent letters (*temps*, *nids*, etc). That way, dyslexics and poor readers cannot use contextual cues - a commonly used strategy to compensate for reading difficulties.

For each child, we computed the Alouette reading fluency and accuracy before and after each training phase (4 times in total). Fluency is defined as the number of words correctly read per minute and accuracy is defined as the percentage of words correctly read per words read in total (correctly + incorrectly read). For each child and each phase, we computed the *change* for reading fluency and accuracy as the difference between the score before and the score after each training.

### Evaluation of meaningful text reading: Monsieur Petit

The Monsieur Petit test is a 274-word-long story that children had to read aloud within a one-minute time limit. This test is used to assess reading skills in French reader and is standardized for children from the end of 1^st^ grade to the 7^th^ grade. Compared to the Alouette test, it is possible to use a contextual strategy when reading. The text is presented in a sheet of paper (font Times New Roman, size 12). Fluency is defined as the number of words correctly read per minute and accuracy is defined as the percentage of words correctly read per words read in total
