# Supplementary material for: A crowding free digital interface to help French-speaking children learn to read
Source: PLoS One. 2025 Jun 25;20(6):e0323623. doi: 10.1371/journal.pone.0323623 (PMC12193705; doi:10.1371/journal.pone.0323623)
Supplement: S1 File — S1.Text. Testing materials. S2.Text. Comparison of the experimental group (Group 1 & Group 2) vs. control group (children in the same class as the experimental group but who did not participate in the training sessions). The active control group was trained with the classical school curriculum by their teacher (n = 4 classes). Teacher participation was voluntary. The 4 teachers who participated were equally experienced (mid-to-late stages of careers). All children were assessed by teachers on the French National reading evaluations, in September and January, and by the research team on reading evaluations (pseudoword reading and meaningful text reading) in June. Only one teacher agreed to share the data from his class on the national evaluation (n = 24, 8 in the control group and 16 in the experimental group).S3.Text. Digital saccade as a function of fluency level during pseudowords reading. S4.Text. Digital saccades as a function of reading fluency performance for meaningless text (Alouette). S5.Text. Finger movement as a function of reading fluency performance for meaningful text (Monsieur Petit). S1.Table. Training stimuli. Overall structure and choice of pseudowords stimuli. In each phase, 20 monosyllabic, 60 bisyllabic and 40 trisyllabic pseudowords were introduced. The complexity of each pseudowords ranged from 1) simple graphemes-simple syllables (CV structure) to 2) complex graphemes-simple syllables (CV structure) and finally 3) complex syllables (CVC or CCV structure). S2.Table. Reading results of control study. Mean and SD for each reading-related skill and each modality (Paper or tablet) were presented, along with p-value of two-sample t-test. S1.Fig. Data of finger kinematics were collected during 6 training sessions (at phase 1 for Group 1 and phase 2 for Group 2). Within each group, children were divided into good or bad decoders based on their decoding score using BELO test. S2.Fig. Change in digital saccade variables according to text decoding level [file pone.0323623.s001.zip › Supplementary_info/S5.Text.docx]

# S5. Finger movement as a function of reading fluency performance for meaningful text (Monsieur Petit)

### Saccade speed

ANOVA-like results from the LMM for *digital saccade speed* showed significant main effect of Meaningful text level (F(1,46)=5.780, p=0.020): good readers of meaningful text made faster saccades than poor ones (good - poor: b=12.365, SE=5.143, t=2.404). There was also an interaction between Session x Meaningful text level (F(5,85144)=64.351, p<0.001) and a triple interaction between Phase x Session x Meaningful text level (F(5,85148)=14.586, p<0.001).

### Saccade length

ANOVA-like results from the LMM for *digital saccade length* showed significant main effect of Meaningful text level (F(1,46)=4.574, p=0.038): good readers made longer saccade than poor readers (good - poor: b=1.448, SE=0.677, t=2.139). There was also an interaction between Session x Meaningful text level (F(5,85143)=55.771, p<0.001) and a triple interaction between Phase x Session x Meaningful text level (F(5,85145)=7.874, p<0.001).

### Fixation duration

No significant main effect of Meaningful level was observed on *digital fixation duration* (F(1,46)=2.230, p=0.142). A significant interaction effect of Session x Meaningful text level (F(5,135515)=6.448, p<0.001) and a triple interaction between Phase x Session x Meaningful text level: (F(5,135515)=6.874, p<0.001) was observed.

### Proportion of regressive saccades

No effect of Meaningful text level was observed (F(1,46)=0.673, p=0.416).
